# Prehospital clinical practice guidelines for unintentional injuries: a scoping review and prioritisation process

**DOI:** 10.1186/s12873-023-00794-x

**Published:** 2023-03-14

**Authors:** Desmond Kuupiel, Nasreen S. Jessani, Jody Boffa, Celeste Naude, Emmy De Buck, Philippe Vandekerckhove, Michael McCaul

**Affiliations:** 1 Division of Epidemiology and Biostatistics, Department of Global Health, Faculty of Medicine & Health Science, Stellenbosch University, Cape Town, 7530 South Africa; 2 Centre for Evidence-Based Health Care, Division of Epidemiology and Biostatistics, Department of Global Health, Faculty of Medicine & Health Science, Stellenbosch University, Cape Town, 7530 South Africa; 3 Faculty of Health Sciences, Durban University of Technology, Durban, 4001 South Africa; 4 Department of Public Health Medicine, School of Nursing and Public Health, University of KwaZulu-Natal, Durban, 4001 South Africa; 5 Department of International Health, Johns Hopkins Bloomberg School of Public Health, Baltimore, USA; 6 The Aurum Institute, Johannesburg, South Africa; 7 Centre for Evidence-Based Practice, Belgian Red Cross, Motstraat 42, 2800 Mechelen, Belgium; 8 Department of Public Health and Primary Care, Leuven Institute for Healthcare Policy, KU Leuven, Kapucijnenvoer 35 block D, 3000 Leuven, Belgium; 9 Cochrane First Aid, Motstraat 42, Mechelen, Belgium; 10 Belgian Red Cross, Motstraat 42, 2800 Mechelen, Belgium

**Keywords:** Injuries, Prehospital care, Clinical practice guidelines, Scoping review, Prioritisation

## Abstract

**Background:**

Globally, millions of people die and many more develop disabilities resulting from injuries each year. Most people who die from injuries do so before they are transported to hospital. Thus, reliable, pragmatic, and evidence-based prehospital guidance for various injuries is essential. We systematically mapped and described prehospital clinical practice guidelines (CPGs) for injuries in the global context, as well as prioritised injury topics for guidance development and adolopment.

**Methods:**

This study was sequentially conducted in three phases: a scoping review for CPGs (Phase I), identification and refinement of gaps in CPGs (Phase II), and ranking and prioritisation of gaps in CPGs (Phase III). For Phase I, we searched PubMed, SCOPUS, and Trip Database; guideline repositories and websites up to 23^rd^ May 2021. Two authors in duplicate independently screened titles and abstract, and full-text as well as extracted data of eligible CPGs. Guidelines had to meet 60% minimum methodological quality according to rigour of development domain in AGREE II. The second and third phases involved 17 participants from 9 African countries and 1 from Europe who participated in a virtual stakeholder engagement workshop held on 5 April 2022, and followed by an online ranking process.

**Results:**

Fifty-eight CPGs were included out of 3,427 guidance documents obtained and screened. 39/58 (67%) were developed de novo compared to 19 that were developed using alternative approaches. Twenty-five out of 58 guidelines (43%) were developed by bodies in countries within the WHO European Region, while only one guideline was targeted to the African context. Twenty-five (43%) CPGs targeted emergency medical service providers, while 13 (22%) targeted first aid providers (laypeople). Forty-three CPGs (74%) targeted people of all ages. The 58 guidance documents contained 32 injury topics. Injuries linked to road traffic accidents such as traumatic brain injuries and chest injuries were among the top prioritised topics for future guideline development by the workshop participants.

**Conclusion:**

This study highlights the availability, gaps and priority injury topics for future guideline development/adolopment, especially for the African context. Further research is needed to evaluate the recommendations in the 58 included CPGs for possible adaptation to the African context.

**Supplementary Information:**

The online version contains supplementary material available at 10.1186/s12873-023-00794-x.

## Background

Globally, it is estimated that unintentional and violence-related injuries contribute to more than 4 million deaths each year and cause many more cases of disability [1]. In 2019, injuries accounted for approximately 8% of total global mortality [2], which, according to the World Health Organisation (WHO), amounts to 32% more than the number of deaths resulting from malaria, tuberculosis and HIV/AIDS combined [1]. Road traffic injuries alone account for approximately 1.3 million deaths annually [2] and remain the leading cause of death and disabilities worldwide [3]. A further 236 000 people lost their lives to drowning in 2019 [4], and burns are estimated to account for about 180 000 deaths annually [4]. While anyone could be the unfortunate victim of injury, mortality and disabilities tend to be higher amongst those in lower-income groups [1] with the majority of deaths occuring before patients reach definitive care, especially in low- and middle-income countries (LMICs) [5, 6]. To this end, prehospital care of injuries is critical to reduce deaths and disabilities.

Prehospital care generally refers to care provided outside the hospital (scene of injury, home, school, disaster area, incidence scene, ambulance environment or other location) until the patient arrives at a formal health care facility capable of providing definitive care [7–9]. Generally, the model of out-of-hospital emergency care (OHEC) systems consist of two tiers [10]. That is, first responder care and community-based transportation (Tier-one OHEC systems); and prehospital care, transport and emergency medical services (Tier-two OHEC systems). To ensure patients injured in the prehospital setting receive effective care that improve patient outcomes and is contextually acceptable, the availability of transparent, reliable and evidence-based clinical practice guidelines (CPGs) play an essential role.

Clinical practice guidelines are statements that include recommendations intended to optimise patient care that are informed by a systematic review of the evidence [11, 12]. A variety of end-user documents such as algorithms and protocols also exist which are often confused with CPGs. Neverheless, end-user documents need to be informed by high quality, relevant and up to date CPGs. The development of new (de novo) CPGs involves a number of steps including i) identifying and refining the topic, ii) convening and running a guideline development group, iii) assessing evidence from systematic reviews, iv) translating evidence into recommendations and v) external review. Methods for developing CPGs de novo are well established [13]. However, developing new CPGs from scratch is neither efficient nor a responsible allocation of limited resources when existing guidelines can be adopted or adapted to meet contextual needs. Guideline adaptation methods have also made clear progress [14], including in prehospital care [15], and is an attractive approach where high-quality CPGs exist.

Knowledge of existing guidelines for prehospital care of injuries is essential to identify gaps and inform next steps, yet review studies describing evidence on CPGs for injuries in a prehospital setting are limited. Scoping reviews can map the available evidence in a field of interest and identify the types of evidence available and knowledge gaps in the literature [16]. A previous scoping review focused on any prehospital guidance specific to SSA, including end-user documents such as protocols and algorithms, with overall very low methodological quality [17]. Thus, the current study a) mapped and described global CPGs for unintentional injuries in a prehospital care setting to inform priorities, gaps and next steps, b) engaged with key stakeholders to identify gaps in CPGs relevant to the African context, and c) shortlisted key CPG gaps for prioritisation.

## Methods

We conducted the study in three sequential phases. Phase I involved a scoping review guided by the Arksey and O’Malley’s methodological framework [22, 23]. Phase II involved identification and refinement of gaps in CPGs; and phase III involved ranking and prioritisation of CPGs. Virtual stakeholder engagement and online ranking were the methodological strategies applied in phases II and III. These methods for phases II and III were drawn upon similar processes for gap identification and prioritisation [17–21].

### Phase I: Scoping review

#### Identifying the review question and study eligibility criteria

We conducted a systematic scoping review to answer the question “What CPGs for injuries in the prehospital care setting have been developed in the past 10 years?”. We used Arksey and O’Malley’s methodological framework for conducting the scoping review [22, 23], and the results are reported in line with the preferred reporting items for systematic reviews and meta-analysis extension for scoping reviews (PRISMA-ScR) [24]. We included CPGs that met the eligibility criteria defined in Table 1.

**Table 1:** Eligibility criteria

Question domains	Inclusion criteria	Exclusion criteria
Population	Individual (infant, children, adult) with unintentional injuries resulting from traffic collisions, drowning, poisoning, fire, falls, others	Individuals with intentional injuries or self-harm such as suicide. CPGs focused only on the mental or psychological-related aspect of injuries or violence-related injuries. Emergencies due to chronic conditions such diabetic and cardiovascular diseases, asthma, obstetric emergencies, and others alike
Concept	Prehospital care: this refers to First Aid and/or emergency medical interventions feasible outside the hospital. That is, CPGs for first responders, basic prehospital care, and advanced prehospital care of injuries	
Setting	Out-of-hospital settings such as incident scene, disaster area, or ambulance environment	Excluded clinic, hospital, refugee, or military settings
Language	All languages	
Publication type	Clinical practice guidelines	Excluded primary research, systematic reviews and other reviews
Time	Publications within 10 years: between 2011 to 2021	
Guideline quality	CPGs that score a minimum of 60% on Domain 3 of AGREE II tool	

#### Identifying relevant guidelines

To identify relevant evidence-based CPGs, we searched MEDLINE (OVID), SCOPUS, and CINAHL databases, and guideline clearinghouses (Scottish Intercollegiate Guidelines Network, Trip, and Guidelines International Network) published between January 2011 and December 2021in any language. We developed the search strategy in consultation with an information specialist who executed the searches (Supplementary file 1). In addition, we manually screened 56 guideline repositories and key organisations’ websites (national and international), such as the WHO, National Institute for Health and Care Excellence, Australian and New Zealand Resuscitation Council, and other prehospital professional associations. All search results were imported into an EndNote Library for compilation and citations management.

#### Guideline selection/ quality appraisal

Title and abstract, and full-text screening was done independently in duplicate (DK and JB) using an a priori developed and piloted eligibility form, with disagreements being resolved by a third reviewer (MM). Ineligible full-texts were excluded with reasons. To ensure accountability of the document selection process, the screening and selection results were reported according to PRISMA [19]. We evaluated all full-text guidelines meeting initial inclusion criteria according to Domain 3 (Rigour of Development) of the AGREE II-tool [25]. The quality appraisal was performed by two investigators independently (DK and JB). To ensure synthesis of only high quality CPGs, we performed data extraction and analysis only on CPGs that met the threshold of 60% on Domain 3.

#### Charting the data

We extracted data using a form designed in Microsoft Excel, which was piloted by two authors (DK and JB) independently on a random sample of included CPGs to standardize interpretation and improve consistency. Primary data extraction was done by a single author (DK) and then reviewed by a second author (JB), with discussion of differences to reach consensus. For each included CPG, we extracted the following information: CPG developer, publication year/date last updated, country where guideline was developed, and WHO Region. In addition, we extracted information about the CPG scope and purpose including title, aim, sub-topic/injury topic, target audience, and target patient population. We further extracted information about the guideline methodology such as development approach (de novo or alternative methods), evidence grading system or tool, and updating information.

#### Collating, summarising, and reporting the results

The extracted data was collated and analysed around five key areas in line with our first aim, namely, guideline selection, characteristics of the included CPGs, quality appraisal, injury topic/type, and intervention category. Analysis was conducted in STATA 14 using simple descriptive statistics, and graphics were developed in Microsoft Excel.

### Phase II: Identification and refinement of gaps in CPGs

To enhance the relevance of this study we sought to ensure that future CPGs meet the needs of the target audiences, as well as reflect the health burden of patient populations based on the opinions/experiences of the target audiences. We, therefore, convened representatives from multiple CPG target audiences in a virtual 3-h stakeholder engagement workshop facilitated by NSJ, MM and CN on 5 April 2022. Participants included first aid responders, paramedics as well as emergency service providers from a range of organisations including the Red Cross, the African Federation of Emergency Medicine (AFEM), National Ambulance Services, Academic departments of Emergency Medicine, Association of Ambulance Professionals, and the Emergency Care Society of South Africa. To ensure an African focus, we sought a balance with respect to gender and local knowledge.

Participants were identified in consultation with experts in the field of FA-PH injury care and with major organisations key to this work (e.g. Belgian Red cross) and those based on the African continent (e.g. AFEM). Participants were purposively selected through consideration of domain, key demographics, and expertise. They were invited to this workshop via email. Second and third choice candidates were also identified in case of non-availability. Permission to digitally capture ideas during Phase II was obtained from the workshop participants.

The objectives were as follows:

- Share findings of the scoping review of existing FA-PH care guidelines for unintentional injury
- Identify additional gaps in CPGs appropriate to the African context
- Agree on next steps for prioritising CPG updates and development.

### Stakeholder Experiences

Stakeholders invited to the workshops generally were working in Africa, had practical experience with first aid and/or prehospital care, and were involved in using and/or implementing CPGs for unintentional injuries. Based on the assumption that first aid responders and prehospital health care workers operate in different circumstances with perhaps different needs, stakeholders from both audiences were invited.

### Identification and prioritisation of CPG gaps

A summary of the scoping review in the form of an infographic was shared with participants in advance of the workshop. During the workshop, a formal presentation of the scoping study served as a springboard for identifying gaps in the CPGs relevant to the African context (also recognizing the heterogeneity within this). Participants were then invited into two audience-specific breakout sessions to identify and discuss CPG topics or interventions that they felt were missing in their contexts.

In the first step, they were each encouraged to think about topic and intervention gaps, as well as context gaps (e.g. where guidelines exist but are not adapted to the African context). This was facilitated using Google Jamboard—a virtual collaborative whiteboard for team co-creation of ideas. Each group was encouraged to brainstorm as many ideas as possible about gaps that they had and add to the jamboard with one idea per post-it note. In the second step, the teams discussed each idea and deliberated its clarity, relevance, and importance using the following four criteria.

1. The existing health burden of the topic gap
2. The impact a guideline on this topic could have on improving quality of care and patient outcomes
3. Lack of clear evidence-based guidelines on the topic
4. Existence of a guideline requiring adaptation in order to have relevance to the African context

Each criteria had a unique code (white circle, purple triangle etc.). Participants were encouraged to mark each refined idea with any two of the four criteria that they deemed most important. This was repeated for each idea until the time in the breakouts sessions was exhausted. The number of ideas that were refined and deliberated varied between the groups and this activity was then further enhanced in Phase III.

### Phase III: Ranking and prioritisation of CPGs

In Phase III of the study, participants were invited to further refine (only the pre-hospital group) and rank the lists (both groups) using Co-Digital (www.codigital.com), an online platform previously used for similar prioritisation exercises in different contexts [19–21]. Participation in this phase was anonymous. Participants were once again encouraged to use the prioritisation criteria mentioned in Phase II. This pairwise ranking process occurred between April 8 and 13, 2022. Participants were recruited as described in phase II.

## Results

### Phase I: Scoping review

#### Guideline selection

**Fig. 1:**
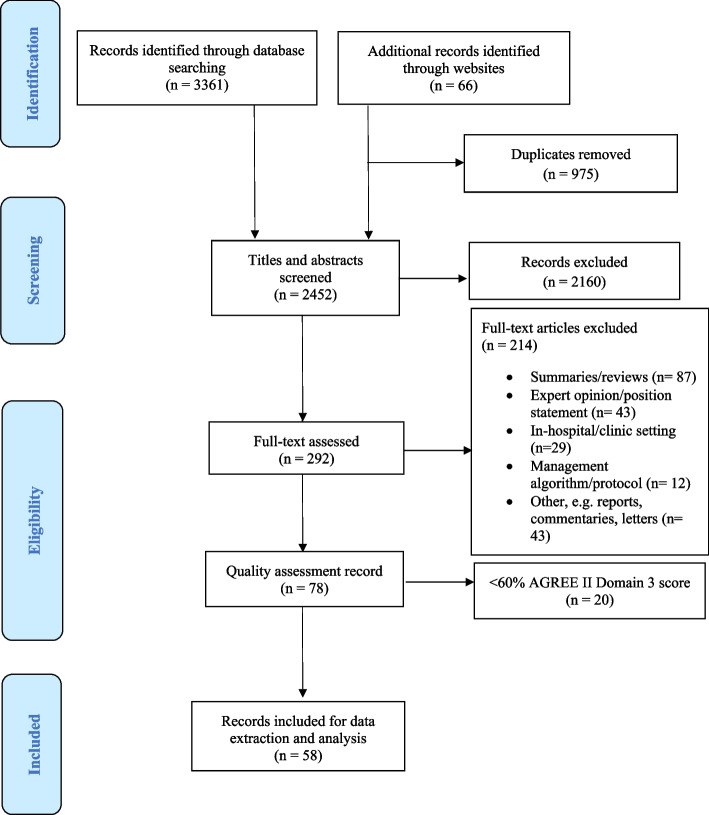
PRISMA flow diagram

We screened 3 427 titles across databases and websites and, after excluding lower quality guidelines (AGREE II domain 3 < 60%), included 58 [26–83] guidelines (Fig. 1). Appendix A presents a list of the excluded documents at the full-text screening phase, whilst a summary of the 20 lower quality guidelines excluded is found in Appendix B.

#### Characteristics of included CPGs and quality appraisal

Of the 58 included CPGs, the majority (*n* = 27, 47%) were developed through international collaborations, and mostly (*n* = 28, 48%) published/updated between 2020 and 2021. Only one included CPG was targeted to the African region [27]. Most target users were clinicians including paramedics: 25 (43%) and an additional 5 (9%) were targeted to paramedics only. With regard to patient population, 43 CPGs (74%) targeted all people (infants, children and adults), while 2 (3.5%) focused specifically on pregnant women. The majority of CPGs was developed de novo (*n* = 39, 67%) and applied the GRADE methodology (*n* = 37, 64%). In most CPGs (*n* = 53, 91.4%), the strength of recommendation(s) was indicated. All details on the characteristics of the included CPGs can be found in Table 2.

**Table 2:** Characteristics of included CPGs

Characteristics	n (%)	Reference
Producer category		
International Collaboration	27 (46.6)	[27, 46, 53, 54, 61–74, 76–83]
Professional Society	16 (27.6)	[28, 29, 31, 32, 34, 36, 38, 41, 44, 45, 47, 49–52, 60]
Government Organisation	13 (22.4)	[26, 30, 33, 35, 37, 42, 43, 48, 55–59]
Academic Institutions	2 (3.4)	[39, 40]
Year of publication / updated		
2011 – 2013	5 (8.6)	[26–30]
2014 – 2016	11 (19.0)	[31–38, 56–59]
2017 – 2019	14 (24.1)	[39–46, 48–50, 55]
2020 – 2021	28 (48.3)	[51–54, 60–74, 76–83]
Target region of guideline		
European Region	25 (43.1)	[26, 34, 38–43, 45, 46, 48, 50–55, 57–59, 81–83]
Western Pacific Region	21 (34.5)	[61–68, 70–74, 76–80]
Region of the Americas	11 (19.0)	[28–33, 35, 36, 47, 49, 60]
African Region	1 (1.7)	[27]
South-East Asian Region (SEAR)		[37]
Target audience/users		
Clinicians (including paramedics)	25 (43.1)	[28, 29, 31, 33–36, 39–42, 44, 45, 50–52, 55, 57–59, 77–80]
Clinicians & laypeople	15 (25.9)	[27, 37, 46, 49, 60–66, 73, 81–83]
Laypeople	13 (22.4)	[26, 43, 53, 54, 67–72, 74, 76]
Paramedics only	5 (8.6)	[30, 32, 38, 47, 48]
Target patient population		
Infants, children and adults	43 (74.1)	[26–30, 32–36, 38, 41, 43–46, 49–55, 57–59, 61–74, 76, 81–83]
Adults only	8 (13.8)	[37, 42, 47, 48, 60]
Infants and children only	5 (8.6)	[31, 77–80]
Pregnant women	2 (3.5)	[39, 40]
Development approach		
De novo	39 (67.2)	[26–44, 46–55, 57–60, 68–72, 82, 83]
Alternative	19 (32.8)	[45, 61–67, 73, 74, 76–81]
Evidence grading system or tool		
GRADE	37 (63.8)	[27, 28, 32, 33, 35, 37, 38, 41–44, 47, 48, 50–55, 57–59, 61–67, 70, 74, 76, 81–83]
Grade & National Health and Medical Research Council hybrid	5 (8.6)	[73, 77–80]
American College of Chest Physicians	3 (5.2)	[36, 46, 49]
EAST Primer	3 (5.2)	[29–31]
Oxford Centre for Evidence-Based Medicine	2 (3.4)	[34, 45]
Shekelle, 1999	2 (3.4)	[39, 40]
American College of Cardiology—American Heart Association	1 (1.7)	[60]
National Health and Medical Research Council (Australia)	1 (1.7)	[69]
Scottish Intercollegiate Guideline Network	1 (1.7)	[26]
Other—not specified	3 (6.9)	[68, 71, 72]
Strength of recommendations presented		
Yes	53 (91.4)	[27–29, 31–55, 57–67, 69, 70, 73, 74, 76–83]
No	5 (8.6)	[26, 30, 68, 71, 72]

The mean quality score ± SD of the 58 included CPGs was 79% ± 12. The mean ± SD score for each domain 3 item out of 7 recorded was: systematic methods were used for evidence (6 ± 2); criteria for selecting evidence are clearly described (6 ± 2); strengths/limitations of evidence described (6 ± 2); methods for formulating evidence are described (6 ± 1); health benefits, side effects, and risks considered (6 ± 2); explicit link between recommendations and evidence (5 ± 2); guideline externally reviewed by experts (5 ± 2); and procedure for updating guideline provided (4 ± 4).

#### Injury topics

**Fig. 2:**
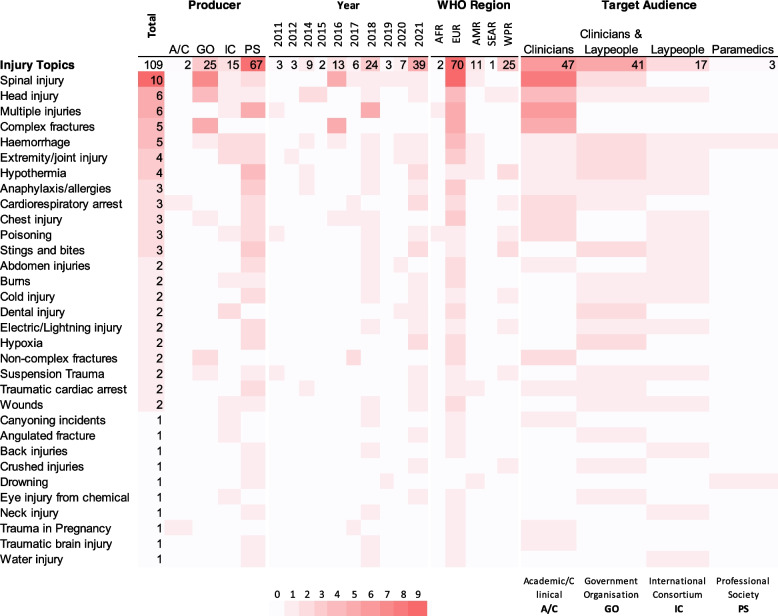
Heat map visualising characteristics of available prehospital care guidance by injury topic

Thirty-two injury topics emanated from the 58 included CPGs. Spinal injury was the most frequent with 10 CPGs, compared to the least common topics, each with 1 CPG: cannoying incidents, angulated fracture, back injuries, crushed injuries, drowning, eye injury from chemicals, neck injury, trauma in pregnancy, traumatic brain injury, and water-related injury. The guidance provided for most of the 32 injury topics was published or updated in 2021, developed by professional societies, developed in or for the European region, and targeted emergency medical service (EMS) providers such as clinicians and paramedics (Fig. 2).

#### Intervention categories

**Fig. 3:**
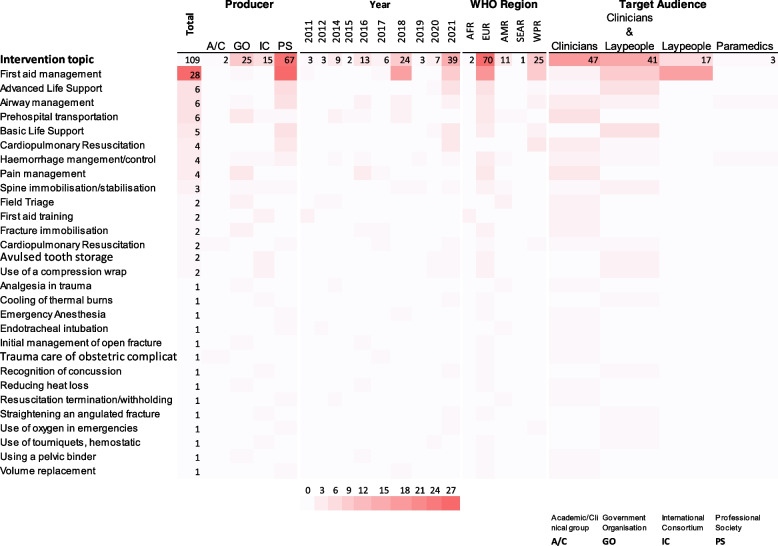
Heat map visualising the distribution of the intervention categories identified from included CPGs

Twenty-nine intervention categories were identified from the 58 included CPGs (Fig. 3). First aid management was the most frequent with 28 CPGs compared to 14 categories each with 1 CPG: analgesia in trauma, cooling of thermal burns, emergency anaesthesia, endotracheal intubation, initial management of open fracture, trauma care of obstetric complication, recognition of concussion, reducing heat loss, resuscitation termination/withholding, straightening an agulated fracture, oxygen use, tourniquet and hemostatic use, use of pelvic binders, and volume replacement.

### Phase II: Identification and refinement of gaps in CPGs

#### Stakeholder overview

A total of 24 stakeholders were invited with 17 participating (8 First Aid, 9 Prehospital care). Participants were from nine African countries (Ghana, Kenya, Tanzania, Uganda, Mozambique, Namibia, South Africa, Zambia, and Zimbabwe) and one European country (Belgium). Those in the First Aid group were all Red Cross practitioners, whereas participants in the Prehospital group included paramedics, as well as emergency service providers from a range of organisations as mentioned in the methods section.

#### Reflections on the CPG scoping review

Discussions around the scoping review presentation included questions around why some CPGs did not feature in the included evidence. Upon being reminded of the scoping review criteria, participants expressed some concerns that perhaps they are using CPGs that did not meet the quality criteria (AGREE II). The only included guideline targeted to the African context was the guideline that resulted in a training manual for first aid providers entitled, “Evidence-based African first aid guidelines and training materials” (AFAM) [27]. Participants discussed that AFAM can be used for identifying CPG gaps for First Aid, specifically for the African context. Gaps in the evidence base to inform CPGs, and challenges with policy implementation were raised by both groups.

#### Brainstorming and refining topics

The brainstorming process resulted in 12 topics and intervention gaps identified by the First Aid Group and 18 identified by the Prehospital group.

The First Aid group highlighted high altitude sickness and mental health as important topics for first aid guidelines. With respect to mental health, it was mentioned that emotional support for the patient and/or a witness to the injury is a critical aspect of the first aid response, and mental health is currently only covered to a limited extent in the AFAM guideline. While these topics do not fall within the scope of this study (unintentional injuries), we wanted to highlight it for future consideration.

Furthermore, the First Aid group noted that the only identified African CPG (AFAM) is already being used widely in practice, and while relevance to the African context was therefore not as much of a concern due to the context specificity of the manual, there exist several implementation challenges, such as subsequent first aid refresher training, and the unique circumstances for first aid in humanitarian settings. Also, participants mentioned gaps in topics, which could be filled by CPGs that our scoping review had found. However, CPGs would then need to be adapted to the African context thereby allowing extension of the manual with context-specific topics. With respect to the Prehospital group, implementation challenges included weaknesses in the broader health systems. These include unclear scopes of practice at the various levels of prehospital care, extent of autonomous decision making by preclinical providers (for instance in situations where patients do not need to be transported or need prolonged field care), legal and ethical considerations of these decisions, weak links to referral processes, suboptimal patient pathways, and vague hospital and patient categorization.

#### Phase III: Ranking and prioritisation of CPGs

Six participants from each of the groups participated in the final ranking exercise. A total of 113 votes across the 18 ideas resulted in 85% consensus (vote agreement) within the Prehospital group. There were 72 votes across 12 ideas with 78% consensus within the First Aid group. Table 3 shows the final prioritised list of CPG topics for the two groups based on participant ranking.

**Table 3:** CPG priority topics emerging from a ranking exercise

Rank	Prehospital Care	Final Score (% of times topic was preferred over the alternative)	First Aid	Final Score (% of times topic was preferred over the alternative)
1	Traumatic Brain Injuries	63.1%	Chest injuries as a result of Road Traffic Accident (punctured lung etc.)	57.4%
2	Haemorrhagic (and neurogenic^b^) shock management	60.9%	Evacuation from a car due to a road traffic accident	56.3%
3	Acute traumatic pain assessment and management	56.0%	Responding to mental/psychological distress as a result of an injury/accident	52.3%
4	Referral pathways for injuries	55.6%	Responding to mental/psychological distress as a result of witnessing an injury/accident	51.3%
5	Mass casualty management	54.0%	Safe evacuation of a drowning victim	50.8%
6	Airway management in the trauma patient	53.1%	Emergency childbirth/delivery	49.5%
7	Palliative (and ethical) care for injury, futility including Do Not Resuscitate (DNR) and non-escalation of care^a^	50.5%	Genital injuries due to Sexual and Gender Based Violence	47.0%
8	Prehospital discharge for minor trauma	50.4%	Removal of protective equipment/clothing from victims of road traffic accidents (eg Helmet, motorcycle boots etc.)	46.7%
9	Trauma in pediatrics	50.0%	Traumatic brain injuries	45.6%
10	Gunshot injury	46.1%	Lightening injury	43.9%
11	Wound care^a^	43.9%	Eye injuries (non-chemical)	38.4%
12	Trauma in pregnant women	43.4%	High Altitude sickness	36.7%
13	Drowning, hypothermia^b^, and resuscitation	43.0%		
14	Infection prevention and control^a^	42.8%		
15	Spinal motion restriction and extrication	41.9%		
16	Burns	41.6%		
17	Geriatric trauma	38.0%		
18	Infectious disease and public health emergencies	36.1%		

^a^Text with asterisk (^a^) denotes topics that need further refinement, Text with superscript hashtag (^b^) denotes edits made by respondents to enhance clarity of the topic

The range of scores was wider for the Prehospital group (36.1%—63.1%) indicating more definitive priorities for this group. For the First Aid group, the distribution was much narrower (36.7%—57.4%) indicating that the relative importance of each topic did not differ greatly, and all topics were of high value.

As expected, the needs from the two groups varied as depicted in Table 3. Similarly, where there were overlapping topics such as traumatic brain injury, drowning, and chest injury /airway management, the relative priority of CPGs for these differed between the groups.

While the Prehospital group was invited to further refine several of the topics to enhance clarity, many of these remained vague or need further unpacking. These topics are denoted in red text. For instance, “wound care” could have been further clarified on whether the needs are for sharp object wounds, burn wounds or infected wounds. Similarly, “Palliative (and ethical) care for injury, futility including DNR and non-escalation of care” might have been separated and clarified. Only one respondent suggested edits, and no one voted or verified those suggested edits. The additions to the original are denoted in green text. For example, “drowning and resuscitation” was edited to “drowning, hypothermia and resuscitation.” With no votes on the edit, the original remained in the list with votes continuing on the original framing. It is worth noting that this group had a large list of topics affiliated with trauma and specifically for various groups (such as for children, for pregnant women, and for the elderly). CPGs relevant to health systems issues such as ethics and referrals ranked within the top ten.

Road traffic injuries were the highest priorities for the First Aid group with four of the top five items related to these. As noted earlier, topics such as mental health responses appear to be extremely important to this group. Unique to this group is also the need to address “genital injuries as a result of sexual and gender based violence” – an issue with rising acknowledgement on the continent, particularly during the COVID-19 pandemic.

## Discussion

This study comprised a three phase process: a scoping review, a stakeholder engagement process, and a prioritization process to identify prehospital CPGs for unintentional injuries. In Phase I we identified 58 CPGs containing 32 injury topics and 29 intervention categories. However, based on this study’s eligibility criteria, there were limited high quality CPGs relevant to the African setting.

Our findings are similar to a previous scoping review that described and appraised prehospital care guidance documents in sub-Saharan Africa which identified 51 guidance documents (mainly protocols and algorithms), of which the majority lacked a trustworthy and transparent evidence foundation with no linkage to a high-quality parent CPG [17]. Our review applied a minimum quality threshold for included prehospital CPGs, ensuring a certain methodological standard. Interestingly, this resulted in only one CPG targeting SSA highlighting the lack of high-quality CPGs for the region and prehospital care.

Our review highlights various reporting and methodological challenges prevalent in prehospital guidance. Firstly, there is poor linkage or reporting of prehospital end-user documents to their parent CPGs or methods. Secondly, high-quality trustworthy guidance is lacking in regions where the burden of injuries is most severe and lastly, guideline adaption of priority topics in prehospital care is still underutilised. Guideline adaptation represents a resource and time efficient method to improve current guidelines to meet methodological standards and provide trustworthy recommendations especially of high priority topics such as Traumatic Brain Injuries, Haemorrhagic and neurogenic shock management, and Chest injuries identified by our stakeholder engagement for SSA. Nonetheless, CPGs must be appropriate for the environment in which they will be used. The burden of injuries in terms of its prevalence, deaths and disability is much higher in LMICs including Africa [1], yet this study found that the majority of the CPGs identified were mostly from European countries and the USA. This has implications towards achieving the sustainable development goal targets linked to injury such as SDG 3.6 and 5.2 in the WHO African Region and other LMIC regions, if not addressed [84]. Resultantly, adapting recommendations from current CPGs could be essential and a more cost-effective and resource-efficient option than creating new CPGs for the African context. CPG developers can employ a local adaptation framework if possible, such as the one created by the ADAPTE Working Group, to create locally appropriate CPGs based on preexisting CPGs [85], especially for high priority injury topics identified as part of the stakeholder engagement. Although the two participant groups identified different topics, there was some overlap that may aid in prioritization for future CPG development or adolopment, such as traumatic brain injury, drowning, and chest injury /airway management.

### Implications for the First Aid providers

The narrow distribution in the scoring of the topics identified by the First Aid group indicates that respondents would appreciate any of these gaps to be filled, as they are ranked similarly in their importance. First Aid training organisations therefore should consider a) adapting CPGs for priority areas identified in the scoping study into the AFAM; b) commissioning the development of African-relevant CPGs where topical gaps have been identified; and c) responding to challenges mentioned by stakeholders that refer to training.

### Implications for Emergency Medical Care providers

These participants consisted of non-physician trained emergency responders, as well as emergency medical personnel. The circumstances within which these responders operate and the resources available to them are different to those for First Aid administrators, which may require tailored CPGs or at least different considerations for each audience. Our study provides a prioritized list of CPGs for Prehospital providers with a fairly clear top five. It would behoove prehospital care groups such as clinicians and paramedics to therefore a) consider this list for creation of Africa-relevant CPGs to fill the identified gaps; b) clarify some of the larger and broader topics in order to ensure stakeholder needs are being met; c) work with other stakeholders to address the health systems components of prehospital care that impact effective emergency response and care (e.g. referrals and decision making); and d) ensure appropriate dissemination, training and use of existing as well as new CPGs.

### Strengths and limitations

The existence of virtual collaboration tools such as Google Jamboard (PhaseII) and Codigital (Phase III) permitted us to convene a geographically dispersed group for discussions and deliberations. Phase II therefore supported synchronous engagement, tailored discussions for the two groups, and communal brainstorming. While this may have been influenced somewhat by group think, the benefits of having a formal scoping review precede the brainstorming session include an evidenced-based starting point for discussion. The asynchronous pair-wise ranking process in Phase III allowed participants to deliberate between two choices at a time rather than simple ranking processes, thereby providing a more nuanced prioritization within the list proposed.

Despite these strengths, the study also has limitations. This scoping review’s data extraction (unit of analysis) did not include recommendations within guidelines. Knowledge of such recommendations may be essential to facilitate adoption to context. Therefore, we recommend further research at the recommendation-level to serve as a resource for guideline users and developers alike. A limitation of this study is our quality appraisal which assessed the CPGs using only one (domain 3) out of the six AGREE-II domains. Certain categories within Domain 3 disproportionately weighed into decisions to include. In particular, providing information on future updating (a more or less binary response), was scored out of 7, meaning CPGs that did not mention updating lost several points on their total score. Some full-text CPGs may have been incorrectly omitted in instances where CPG development was described elsewhere, but not referenced within the CPG itself. Possibly, a few more CPGs would have been judged to be of high quality if all the six domains were considered. In Phase III of our study, unfamiliarity with the Codigital platform limited use of the refinement options within the Prehospital care group, leaving us with some unclear topics. Furthermore, the process did not distinguish the unique priorities for each country, but rather provided an aggregate score for respondents across the nine African countries who voted. However, given the similarity of topics that appeared across respondents in Phase II, we are confident that any of the CPGs developed as a result of this study will be of benefit to all. Despite these limtations, it is worth mentioning that, to the best of our knowledge, this review is the first of its kind in concept and context. The addition of a prioritisation process lends further value to future CPG development and adolopment. We further encourage CPG development organisations to peruse this study to guide next steps in CPG development that would speak to the priorities of the First Aid and Prehospital care groups in Africa.

## Conclusion

Based on this study’s eligibility criteria, 109 CPGs have been developed for prehospital care of patients in the past 10 years. The CPGs identified has a variety of injury topics and linked interventions from the CPGs that can be considered by prehospital guideline developers for adaptation. We identified regional priority topics relevant to first aid (chest injuries, evacuation from a car, and mental/psychological distress due to a road traffic accident, safe evacuation of a drowning victim, and genital injuries due to sexual and gender based violence); and prehospital care (traumatic brain injuries, haemorrhagic and neurogenic shock management, acute traumatic pain assessment and management, referral pathways for injuries, and mass casualty management). Further research is needed to match priority topics with individual recommendations from high-quality CPGs to map feasibility and need for guideline adaptation or de novo development.

## Supplementary Information

**Additional file 1:** **Supplementary file 1.** Databases search strategy.

**Additional file 2:** **Appendix A.** List of documents at full-text screening stage

**Additional file 3: Appendix B. **Summary of low quality CPGs excluded.

## Data Availability

All data for Phase I have been presented in the form of references. Data for Phases II & III has cannot be shared because they have some participant identifiers. The corresponding author (Dr Desmond Kuupiel) can be contacted for the data if needed.
