# Supplementary material for: Prehospital clinical practice guidelines for unintentional injuries: a scoping review and prioritisation process
Source: BMC Emerg Med. 2023 Mar 14;23:27. doi: 10.1186/s12873-023-00794-x (PMC10010958; doi:10.1186/s12873-023-00794-x)
Supplement: Supplementary file 1 — Additional file 1: Supplementary file 1. Databases search strategy. [file 12873_2023_794_MOESM1_ESM.docx]

**Supplementary file 1: Databases search strategy**

Database: Ovid MEDLINE(R) and In-Process, In-Data-Review & Other Non-Indexed Citations <1946 to May 18, 2021>

Search Strategy:

--------------------------------------------------------------------------------

1 guideline/

2 guideline*.tw.

3 practice guideline/

4 1 or 2 or 3

5 accident prevention/ or accidental falls/ or accidents, aviation/ or biohazard release/ or chemical hazard release/ or accidents, home/ or accidents, occupational/ or accidents, traffic/ or drowning/ or radioactive hazard release/

6 exp Burns/

7 *Poisoning/

8 poisoning/ or *arsenic poisoning/ or exp "bites and stings"/ or *carbon tetrachloride poisoning/ or *fluoride poisoning/ or *gas poisoning/ or *heavy metal poisoning/ or *organophosphate poisoning/ or *plant poisoning/

9 Carbon Monoxide Poisoning/

10 exp Disasters/

11 exp "Wounds and Injuries"/

12 accidental injuries/ or arm injuries/ or athletic injuries/ or back injuries/ or "bites and stings"/ or crush injuries/ or joint dislocations/ or drowning/ or electric injuries/ or fractures, bone/ or frostbite/ or hand injuries/ or hip injuries/ or lacerations/ or leg injuries/ or multiple trauma/ or shoulder injuries/ or "sprains and strains"/ or tooth injuries/

13 (injury or injuries or trauma*).tw.

14 (poisoning* or burns or "car crash*" or accidents or "road crash*" or drown* or "accidental fall*" or "bone fracture*").tw.

15 sprains.mp.

16 dislocation*.tw.

17 Anaphylaxis/ or anaphyla*.mp.

18 Lightning Injuries/ or Lightning/

19 (concussion or concussed).tw.

20 choking.mp.

21 crashes.mp.

22 5 or 6 or 7 or 8 or 9 or 10 or 11 or 12 or 13 or 14 or 15 or 16 or 17 or 18 or 19 or 20 or 21

23 early management.mp.

24 emergency medical services.tw. or Emergency Medical Services/

25 first aid.tw. or First Aid/

26 (prehospital or pre-hospital).tw.

27 (out of hospital or out-of-hospital).tw.

28 field triage.mp.

29 pre-admission.mp.

30 patient transport*.mp. or Ambulances/ or "Transportation of Patients"/

31 23 or 24 or 25 or 26 or 27 or 28 or 29 or 30

32 4 and 22 and 31

33 limit 32 to yr="2010 -Current"

34 (army or military or defense or refugee* or humanitarian or Covid*).ti.

35 33 not 34

36 limit 35 to humans

***************************

SCOPUS

( ( ( TITLE-ABS-KEY ( "emergency medical service*" ) ) OR ( TITLE-ABS-KEY ( "first aid" ) ) OR ( TITLE-ABS-KEY ( "early management" ) ) OR ( TITLE-ABS-KEY ( "patient transport*" ) ) OR ( TITLE-ABS-KEY ( "out of hospital" ) ) OR ( TITLE-ABS-KEY ( out-of-hospital ) ) OR ( TITLE-ABS-KEY ( ambulance* ) ) OR ( TITLE-ABS-KEY ( pre-admission ) ) OR ( TITLE-ABS-KEY ( field AND triage ) ) OR ( TITLE-ABS-KEY ( prehospital OR pre-hospital ) ) ) AND ( ( TITLE-ABS-KEY ( guideline* ) ) AND ( ( TITLE-ABS-KEY ( choking ) ) OR ( TITLE-ABS-KEY ( lightning AND injuries ) ) OR ( TITLE-ABS-KEY ( sprains OR dislocation* OR concussion* ) ) OR ( TITLE-ABS-KEY ( drowning ) ) OR ( TITLE-ABS-KEY ( "car crash*" OR "traffic accidents" OR "road crash*" ) ) OR ( TITLE-ABS-KEY ( wounds ) ) OR ( TITLE-ABS-KEY ( natural AND disasters ) ) OR ( TITLE-ABS-KEY ( trauma OR traumas ) ) OR ( TITLE-ABS-KEY ( burns OR poisoning ) ) OR ( TITLE-ABS-KEY ( accidents OR injuries OR injury ) ) ) ) ) AND NOT ( TITLE ( army OR military OR defense OR refugee* OR humanitarian OR covid* ) ) AND ( LIMIT-TO ( SUBJAREA , "MEDI" ) OR LIMIT-TO ( SUBJAREA , "NURS" ) OR LIMIT-TO ( SUBJAREA , "HEAL" ) OR LIMIT-TO ( SUBJAREA , "SOCI" ) ) AND ( LIMIT-TO ( DOCTYPE , "ar" ) ) AND ( LIMIT-TO ( EXACTKEYWORD , "Human" ) OR LIMIT-TO ( EXACTKEYWORD , "Humans" ) ) AND ( LIMIT-TO ( PUBYEAR , 2021 ) OR LIMIT-TO ( PUBYEAR , 2020 ) OR LIMIT-TO ( PUBYEAR , 2019 ) OR LIMIT-TO ( PUBYEAR , 2018 ) OR LIMIT-TO ( PUBYEAR , 2017 ) OR LIMIT-TO ( PUBYEAR , 2016 ) OR LIMIT-TO ( PUBYEAR , 2015 ) OR LIMIT-TO ( PUBYEAR , 2014 ) OR LIMIT-TO ( PUBYEAR , 2013 ) OR LIMIT-TO ( PUBYEAR , 2012 ) OR LIMIT-TO ( PUBYEAR , 2011 ) OR LIMIT-TO ( PUBYEAR , 2010 ) )

Cinahl (Interface – EBSCOhost)

| **#** | **Query** |
| --- | --- |
| S27 | S25 NOT S26 |
| S26 | TI army or military or defense or refugee* or humanitarian or Covid* |
| S25 | S1 AND S23 |
| S24 | S1 AND S23 |
| S23 | S13 AND S22 |
| S22 | S14 OR S15 OR S16 OR S17 OR S18 OR S19 OR S20 OR S21 |
| S21 | (MH "Ambulances") OR "ambulance*" |
| S20 | TX patient transport* |
| S19 | TX pre-admission |
| S18 | TX field triage |
| S17 | TX (out of hospital or out-of-hospital) |
| S16 | TX first aid |
| S15 | "emergency medical services or ems or prehospital or pre-hospital or paramedic" OR (MH "Emergency Service") |
| S14 | TX early management |
| S13 | S2 OR S3 OR S4 OR S5 OR S6 OR S7 OR S8 OR S9 OR S10 OR S11 OR S12 |
| S12 | TX concussion or concussed or choking |
| S11 | TX lightning |
| S10 | TX Anaphylaxis/ or anaphyla* |
| S9 | TX sprains or dislocation* |
| S8 | TX drowning |
| S7 | TX road traffic accident* |
| S6 | ""car crash*" or accidents or "road crash*" or drown* or "accidental fall*" or "bone fracture*"" |
| S5 | TX wounds |
| S4 | TX natural disasters or tsunamis or floods or drought or wildfire or earthquake or tornado or hurricane or snowstorm |
| S3 | TX ( burns or burn injury or burns trauma or major burns ) OR TX poisoning OR TX ( bites or stings ) |
| S2 | TX accidents or injuries |
| S1 | TX guideline* |

TRIP database:

injury trauma guidelines pre-hospital
