# Supplementary material for: Prehospital clinical practice guidelines for unintentional injuries: a scoping review and prioritisation process
Source: BMC Emerg Med. 2023 Mar 14;23:27. doi: 10.1186/s12873-023-00794-x (PMC10010958; doi:10.1186/s12873-023-00794-x)
Supplement: Supplementary file 3 — Additional file 3: Appendix B. Summary of low quality CPGs excluded. [file 12873_2023_794_MOESM3_ESM.docx]

***Summary of the 20 CPGs excluded with < 60% AGREE-II domain 3 score***

The mean ± SD AGREE-II domain 3 score of these 20 CPGs was 44% ± 9. The mean (± SD) score for each domain 3 item out of 7 recorded was: systematic methods were used for evidence (3 ± 2); criteria for selecting evidence are clearly described (4 ± 2); strengths/limitations of evidence described (3 ± 2); methods for formulating evidence are described (4 ± 2); health benefits, side effects, and risks considered (4 ± 2); explicit link between recommendations and evidence (4 ± 2); guideline externally reviewed by experts (4 ± 1); and procedure for updating guideline provided (3 ± 3). All 20 CPGs were published between 2011 and 2021, with the majority published in the last 5 years. Of the 20 CPGs, 6 (30%) were developed by institutions in the USA, 4 (20%) from Australia and New Zealand, 2 (10%) from France, and the remainder 8 (40%) from South Africa, Malaysia, Singapore, Germany, Canada, Switzerland, Italy, and Japan with 1 (5%) CPG each. Twelve injury topics were identified from the 20 excluded CPGs with spinal injury and head injury appearing most frequently (3 each) (Figure 4). Targeted users of the guidance provided by these 20 CPGs included EMS providers only (n = 8, 40%), First Aid providers (n = 5, 25%), First Aid and EMS providers (n = 4, 20%), Terrestrial rescue service providers, Athletic trainers and others athletic healthcare providers, and Eductors of healthcare providers with 1 (5%) each.

**Supplementary figure 1:** Injury topics identified from the 20 excluded CPGs with less than 60% AGREE-II domain 3 score
